# Heparan sulfate proteoglycan-dependent neutrophil chemotaxis toward PR-39 cathelicidin

**DOI:** 10.1186/1476-9255-3-14

**Published:** 2006-11-02

**Authors:** Angela Djanani, Birgit Mosheimer, Nicole C Kaneider, Christopher R Ross, Giovanni Ricevuti, Josef R Patsch, Christian J Wiedermann

**Affiliations:** 1 Laboratory of Medical Intensive Care, Division of General Internal Medicine, Department of Medicine, Medical University of Innsbruck, Anichstrasse 35, A-6020 Innsbruck, Austria; 2 Department of Anatomy and Physiology, College of Veterinary Medicine, Kansas State University, Coles Hall 228, 1600 Denison Avenue, Manhattan, KS 66506-5602, USA; 3 Department of Internal Medicine and Therapeutics, Section of Internal Medicine, University of Pavia, Viale Liberta, I-27100 Pavia, Italy

## Abstract

Cathelicidins are mammalian proteins containing a C-terminal cationic antimicrobial domain. Porcine PR-39 cathelicidin affects leukocyte biology. Mechanisms of action may involve alteration of heparan sulfate proteoglycan-dependent functions in inflammatory cells. It was tested  whether PR-39 affects human neutrophil migration and if such effects involve heparan sulphate proteoglycans. Neutrophils were from forearm venous blood of healthy donors. Migration was tested in modified Boyden chamber assays. Involvement of heparan sulfate proteoglycans was tested by their chemical modification and by the use of specific antibodies. PR-39 induced migration in neutrophils in a concentration dependent manner. Modification of heparan sulfate proteoglycans with sodium chlorate inhibited migration whereas chemotaxis toward the chemoattractant formyl-Met-Leu-Phe was not affected. Removal of heparan sulfates or chondroitin sulfates from the surface of  neutrophils by heparinase or chondroitinase inhibited migration toward  PR-39. In conclusion, antimicrobial PR-39 stimulates human neutrophil chemotaxis in a heparan sulfate proteoglycan-dependent manner. Involvment of  syndecans is likely as both heparinase and chondroitinase were abrogating. Data suggest active participation of heparan sulfate proteoglycans of neutrophils in cathelicidin peptide-mediated regulation of the antimicrobial  host defense.

## Findings

Peptides with *in vitro *antimicrobial activity have been identified from several gene families. Two major antimicrobial peptide families in mammals are the defensins [1] and the cathelicidin peptides [2-4]. It is known that the defensin structure is based on a common beta sheet core, which is stabilized by three disulfide bonds [1,2] but cathelicidins are highly heterogeneous. Their conserved cathelin domain sequence has been used as a genetic probe enabling the discovery of numerous new members of this family [4-10]. Circulating neutrophils, myeloid bone marrow cells and epithelial surfaces are an important source of cathelicidine expression [7,11-14]. The cathelicidin, prolin-arginine-rich 39 peptide (PR-39), first isolated from the porcine small intestine [15] was also identified in porcine neutrophils [16]. Different forms occur and PR-39 isolated from porcine small intestine is sligthly different in composition from that isolated from porcine neutrophils [17]. PR-39 posseses antibacterial activity [18] and has the ability to induce syndecan expression in wounds in animal studies [12]. PR-39 kills bacteria by a mechanism that stops protein and DNA synthesis after a lag period of about 8 min [18]. PR-39 is an inhibitor of neutrophil function in injured mice, is involved in metastatic activity of human tumor cells, and can induce angiogenesis [1-3,19,20]. These observations suggest that the efficacy of PR-39 is not species specific.

Heparan sulfate proteoglycans (HSPG) from endothelium and leukocytes interact with P-selectin, an important adhesion molecule regulating leukocyte adhesion and migration [21]. HSPG localize to granules of myeloid cells including monocytes and neutrophils [22], and expression of mRNA for syndecan core protein has been detected in different types of leukocytes including neutrophils [23,24]. As PR-39 is abundantly expressed in mammalian tissues and is best investigated among cathelicidins, our motivation in this project was to determine if porcine PR-39 affects human neutrophil migration and whether such effects involve HSPG.

Heparinase I and chondroitinase ABC were from Sigma Chemical Corp. (St. Louis, MO, USA). PR-39 was synthesized by solid-phase method with greater than 90% purity [25]. Antibodies against the core-protein of syndecan-4 (D-16) and the ectodomain of this proteoglycan (5G9) were affinity purified goat polyclonal antibodies raised against peptides mapping with the respective regions of the human syndecan-4 protein (both Santa Cruz Inc., Wiltshire, England). According to the manufacturers instructions, suitability of its use has been demonstrated for detection of syndecan-4 as well as for use as control antibodies in siRNA studies. Other reagents not further specified were also from Sigma. Neutrophils were obtained from forearm venous blood of healthy volunteers, anticoagulated with 1.6 mg EDTA/mL of blood. Neutrophil preparation was performed as described [26]. Cell preparations yielded > 95% neutrophils (by morphology in Giemsa stains) and > 99% viability (by trypan dye exclusion). Chemotaxis of neutrophils into cellulose nitrate to gradients of soluble attractants was measured in RPMI 1640/0.5% BSA using a 48-well microchemotaxis chamber (Neuroprobe, Bethesda, MD, USA) in which a 5 μm pore-sized filter (Sartorius, Göttingen, Germany) separates the upper and lower chamber as previously described [26]. When PR-39 was used as attractant, concentrations ranging from 10 mmol/L to 1 nmol/L were tested. As positive control chemotactic agent in the lower chamber fMLP was used. For some experiments cells were pretreated with heparinase I, an enzyme that cleaves highly sulfated regions of heparan sulfate-like glycosaminoglycans at 2-O-sulfated uronic acids, for 50 min. Thereafter cells were washed twice before testing for chemotaxis. For other experiments cells were pretreated with chondroitinase ABC also for 50 min that cleaves chondroitin sulfate side chains of cell surface proteoglycans. Since it is known that sodium chlorate is able to modify proteoglycan sulfation, we tested PR-39 chemotaxis after pre-treatment of cells for 20 min with sodium chlorate. As neutrophil migration toward PR-39 might be mediated via syndecan-4, chemotaxis experiments were performed in the presence of monoclonal antibodies toward the core-protein of syndecan-4 and a side chain of this proteoglycan. Cells were incubated with these antibodies for 20 min, washed twice and allowed to migrate toward PR-39.

Data are expressed as mean and standard error of the mean (S.E.M.). Means were compared by Kruskal-Wallis analysis of variance and by Mann-Whitney u-test for nonparametric samples (Abacus Concepts, Berkley, CA). A difference with p < 0.05 was considered to be significant.

**Figure 1:**
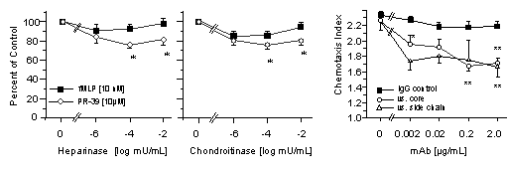
**Effects of heparinase, chondroitinase and anti-syndecan 4 antibodies on PR-39-induced chemotaxis of neutrophils**. Heparinase I (*left panel*) or chondroitinase (*middle panel*) was added to neutrophils. After an incubation (humidified, 37°C/5% CO_2_) period of 50 min, cells were washed twice and chemotaxis assays were performed. PR-39 [10 μmol/L] was used as chemoattractant and fMLP [10 nmol/L] served as control attractant. Data are expressed as percent of medium control (pretreatment with medium), with a distance of migration toward fMLP of 79.8 ± 3.1 μm, towards PR-39 of 66.8 ± 1.3 (n = 4). Statistical analysis: Mann-Whitney U-test versus medium control, *, p < 0.05 after Kruskal-Wallis test, p < 0.05. Effects of antibodies to syndecan-4 core protein or syndecan-4 chain epitopes (*right panel*) were tested by preincubation of neutrophils for 20 minutes (humidified 37°C/5% CO_2_). After washing, cells were allowed to migrate toward PR-39 [10 μmol/L] in modified Boyden chambers using nitrocellulose micropore filters. Isotype matched IgG served as control. Results are given as mean ± SEM of the chemotaxis index, which is the ratio between the distance of migration toward attractant and that toward control. Distance of random migration was 40.0 ± 2.78 (n = 4). Statistical analysis: Mann-Whitney U-test versus no antibody, * p < 0.05 after Kruskal-Wallis test p < 0.05.

To determine whether PR-39 induces human neutrophil chemotaxis, we tested *in vitro *migration of the cells at a wide range of concentrations [10 mmol/L to 1 nmol/L]. PR-39 induced human neutrophil chemotaxis in a concentration dependent manner with a maximum effect at 10 μmol/L (data not shown). To investigate the role of intact HSPG on the surface of neutrophils in PR-39-induced cell migration, neutrophils were pretrated for 50 min with heparinase I or chondroitinase [both, 50 nU/mL to 50 mU/mL] at 37°C, followed by two washing steps. As glypicans carry heparan sulfate side chains but not chondroitin sulfate side chains, whereas syndecans carry both [27], experiments were performed with both heparinase I and chondroitinase. Results showed a concentration-dependent reduction of migration by removal of these two substrates from the cell surface, whereas chemotactic effects of fMLP [10 nmol/L] were not affected (Fig. 1). To investigate the effect of sodium chlorate which is known to modify sulfation of proteoglycans and sulphated proteins in cell culture, neutrophils were pretreated with sodium chlorate [10 mmol/L to 40 mmol/L], washed, and then allowed to migrate towards PR-39. Neutrophil chemotaxis to PR-39 was significantly inhibited by sodium chlorate whereas chemotaxis toward the chemokine IL-8 used as alternative control attractant was not affected (data not shown).

Because PR-39-induced chemotaxis was inhibited by chondroitinase and heparinase I, we suggested the involvement of syndecans but not glypicans by PR-39. Moreover, *in vivo *expression of syndecans has been reported as being affected by PR-39 [12]. Therefore, chemotaxis of neutrophils toward PR-39 was tested in the presence of migration-blocking monoclonal antibodies to syndecan-4 core protein or a syndecan-4 side chain because this pathway was shown to affect the cell's motility. Neutrophils were again pre-treated with either of the two antibodies or an isotype-matched IgG, and then allowed to migrate toward PR-39 [10 μmol/L]. The antibodies specifically inhibited neutrophil migration toward PR-39 (Fig. 1).

In the present study, PR-39 stimulated human neutrophil chemotaxis in a bell-shaped dose-response curve. PR-39 is known to induce chemotaxis in porcine neutrophils [25]. As PR-39 is effective in improving survival in animals models of severe sepsis [28,29]. the observation that PR-39 affects neutrophil function accross different species may be of relevance if PR-39 is further developed for potential use in severe sepsis. In porcine leukocyte chemotaxis, peak responses occurred at 0.5 to 2 micromoles per liter [25] which correlates well with PR-39 effects on human neutrophils (data not shown). This finding suggests that exogenous PR-39 may play a role not only in animal but also in human inflammation.

For LL-37, the only human cathelicidin identified so far, it was proposed that chemotactic responsiveness of leukocytes involves formyl peptide receptor-like 1, and activation of this formyl peptide receptor-like 1 cross-deactivates LL-37 responsiveness [30]. In mast cell responses to cathelicidins, there may be two types of receptors involved, a high affinity receptor responsible for chemotaxis, and a low affinity receptor with undefined function [31]. Detailed biochemical mechanisms or a biochemical characterization of these binding sites, however, remained unknown.

Antithrombin-III, a prototypical glycosaminoglycan ligand, has only recently been identified to exert direct effects on cells of the innate immune system via HSPG [26,32]. Already two decades ago, consensus sequences for glycosaminoglycan recognition were determined as [-X-B-B-X-B-X-] and [-X-B-B-B-X-X-B-X-] where B is the probability of a basic residue and X is a hydropathic residue, which form potential nucleation sites for the recognition of polyanions in proteins [33]. As antimicrobial peptides including defensins and cathelicidins contain consensus sequences for HSPG recognition [34], we further explored the roles of HSPG in mediating PR-39 effects in neutrophil migration. Heparan sulfate chains abound on syndecans and glypicans which can bind a repertoire of proteins. Thereby, HSPG can immobilize the ligands, increase its local concentration, change its conformation, present it to a signalling receptor and enhance the formation of receptor-ligand signaling complexes [35]. Modification of these heparan sulfate chains could influence cell function. To further substantiate the role of HSPG in the response of neutrophils to PR-39, cells were pretreated with the sulfation inhibitor, sodium chlorate [36]. This pretreatment dose-dependently inhibited responiveness of the cells to PR-39. Moreover, of the two families of membrane-bound HSPG, the syndecans, by containing mixtures of the two major types of glycosaminoglycan chains found in animal cells, namely heparan sulfate and chondroitin sulfate, exemplify hybrid proteoglycans. In contrast, the glypicans appear to contain only heparan sulfate chains [37]. To examine if chondroitin sulfate proteoglycans act as putative interaction site for PR-39, neutrophils were treated with chondroitinase before testing cell migration. Data imply that PR-39's effects on cell migration are sensitive to both heparinase I and chondroitinase suggesting that of HSPG, syndecans mediate direct cellular actions of PR-39. By cleaving this specific site enzymatically with heparinase or chondroitinase or by chemically modifying them with sodium chlorate, chemotactic effects of PR-39 were altered. These observations not only confirm the role of HSPG in leukocyte function but also functionally establish that syndecans are involved in signalling effects of the cathelicidin in leukocytes.

The syndecan family of cell surface proteoglycans has been implicated in a number of biological processes, including blood coagulation, cell adhesion, signal transduction and wound repair [38]. Originally found on epithelial cells, syndecans were later shown to be present in several other mesenchyma-derived cell types, including fibroblast, smooth muscle cells, and neutrophils [28].

PR-39 has been shown to interact with a domain within the integrin mediated signaling protein, namly p130(Cas) [39], an assembling molecule of actin filaments which promotes cell movement, cell migration, and cell spreading in fibroblasts [40]. Identification of p130(Cas) as a mediator of focal adhesion kinase-promoted cell migration [41] fits well to its interaction with PR-39, given the finding that syndecan-4 modulates focal adhesion kinase phosphorylation [42] and may be activated by PR-39 as suggested by our observed inhibition of PR-39-induced chemotaxis of neutrophils with antibodies to syndecan-4. This hypothesis, however, requires biochemical confirmation.

The data provided do not permit the conclusion that only syndecan-4 mediates the chemotaxis of human neutrophils to PR-39. Other syndecans or glypicans may also participate in coordinating the chemotactic response, as additional cell surface proteoglycans harboring HS and/or CS might also be involved.

To summarize, besides proposed involvement of formyl peptide receptor-like 1, no signalling pathway for cathelicidins in neutrophils had been identified so far. Our results provide strong evidence for interactions of PR-39 with proteoglycans on the surface of leukocytes. Biochemical and functional tests identify syndecan-4 as a putative acceptor site for PR-39 which contains consensus sequences for glycosaminoglycan recognition. Our findings may be of particular relevance if PR-39 proofs have a therapeutic potential in neutrophil-mediated inflammatory diseases.

## Abbreviations

BSA – Bovine serum albumin

fMLP – formyl-Met-Leu-Phe

HSPG – Heparan sulfate proteoglycan

PR-39 – Prolin-arginine-rich 39 peptide

## Competing interests

The author(s) declare that they have no competing interests.

## Authors' contributions

AD carried out the chemotaxis experiments and drafted the manuscript. BM and NCK carried out the enzyme digestion and antibody inhibition studies, respectively. CRR, GR and JRP participated in the design of the study. CJW conceived of the study, and participated in its design and coordination and helped to draft the manuscript. All authors read and approved the final manuscript.
